# Somatostatin receptor based PET/CT in patients with the suspicion of cardiac sarcoidosis: an initial comparison to cardiac MRI

**DOI:** 10.18632/oncotarget.12799

**Published:** 2016-10-21

**Authors:** Constantin Lapa, Theresa Reiter, Malte Kircher, Andreas Schirbel, Rudolf A. Werner, Theo Pelzer, Carmen Pizarro, Dirk Skowasch, Lena Thomas, Ulrike Schlesinger-Irsch, Daniel Thomas, Ralph A. Bundschuh, Wolfgang R. Bauer, Florian C. Gärtner

**Affiliations:** ^1^ Department of Nuclear Medicine, University Hospital Würzburg, Würzburg, Germany; ^2^ Department of Internal Medicine, University Hospital Würzburg, Würzburg, Germany; ^3^ Comprehensive Heart Failure Center, University Würzburg, Würzburg, Germany; ^4^ Department of Internal Medicine II - Pneumology/Cardiology, University Hospital Bonn, Bonn, Germany; ^5^ Department of Nuclear Medicine, University Hospital Bonn, Bonn, Germany; ^6^ Department of Radiology, University Hospital Bonn, Bonn, Germany

**Keywords:** sarcoidosis, DOTATOC, SSTR, somatostatin receptor, PET

## Abstract

Diagnosis of cardiac sarcoidosis is often challenging. Whereas cardiac magnetic resonance imaging (CMR) and positron emission tomography/computed tomography (PET/CT) with ^18^F-fluorodeoxyglucose (FDG) are most commonly used to evaluate patients, PET/CT using radiolabeled somatostatin receptor (SSTR) ligands for visualization of inflammation might represent a more specific alternative. This study aimed to investigate the feasibility of SSTR–PET/CT for detecting cardiac sarcoidosis in comparison to CMR.

15 patients (6 males, 9 females) with sarcoidosis and suspicion on cardiac involvement underwent SSTR-PET/CT imaging and CMR. Images were visually scored. The AHA 17-segment model of the left myocardium was used for localization and comparison of inflamed myocardium for both imaging modalities. In semi-quantitative analysis, mean (SUV_mean_) and maximum standardized uptake values (SUV_max_) of affected myocardium were calculated and compared with both remote myocardium and left ventricular (LV) cavity.

SSTR-PET was positive in 7/15, CMR in 10/15 patients. Of the 3 CMR^+^/PET^−^ subjects, one patient with minor involvement (<25% of wall thickness in CMR) was missed by PET. The remaining two CMR^+^/PET^−^ patients displayed no adverse cardiac events during follow-up.

In the 17-segment model, PET/CT yielded 27 and CMR 29 positive segments. Overall concordance of the 2 modalities was 96.1% (245/255 segments analyzed). SUV_mean_ and SUV_max_ in inflamed areas were 2.0±1.2 and 2.6±1.2, respectively. The lesion-to-remote myocardium and lesion-to-LV cavity ratios were 1.8±0.2 and 1.9±0.2 for SUV_mean_ and 2.0±0.3 and 1.7±0.3 for SUV_max_, respectively.

Detection of cardiac sarcoidosis by SSTR-PET/CT is feasible. Our data warrant further analysis in larger prospective series.

## INTRODUCTION

Sarcoidosis is a multisystem granulomatous disorder of unknown etiology that can virtually involve all organ systems [1]. Whereas lymph nodes and lungs are most commonly affected, cardiac involvement is present in up to one third of patients [2, 3] and has been reported as the second leading cause of death by sarcoidosis in the United States and the leading cause in Japan [4]. Therefore, early diagnosis and treatment initiation is mandatory. However, diagnosis is often challenging due to the patchy, multifocal pattern of the disease rendering sensitivity of endomyocardial biopsy as low as 20-30% [5]. In clinical routine, the Guidelines of the Japanese Ministry of Health and Welfare (JMHWG) and the Heart Rhythm Society (HRS) serve as a standard for the diagnosis of cardiac sarcoidosis (CS) and include a combination of clinical and imaging findings, such as ECG abnormalities and signs of acute myocardial damage on established cardiac imaging techniques [6, 7]. In terms of cardiac imaging, both cardiac magnetic resonance imaging (CMR) and positron emission tomography/computed tomography (PET/CT) with ^18^F-fluorodeoxyglucose (FDG) have proven its value for CS detection and patient prognostication [2, 8–10]. However, specificity of FDG-PET is hampered by physiologic tracer accumulation requiring dedicated patient preparation [11–13]. As a more specific alternative, feasibility of PET targeting the somatostatin receptor (SSTR) subtype 2 has been demonstrated for imaging atherosclerosis [14–16], myocardial inflammation [17] and CS [18]. The aim of this study was to further assess the feasibility of SSTR–PET/CT for detecting CS in comparison to CMR.

## RESULTS

### Clinical findings

All but one patient (Patient #9) presented with biopsy-proven sarcoidosis. Most commonly, mediastinal und hilar lymph nodes (n=13) and the lungs (n=14) were affected. Other sites of organ involvement included spleen (n=2), liver (n=2), skin (n=2), bone (n=1), parotic glands (n=1), omentum (n=1), uvea (n=1) and the nasal sinuses (n=1).

Cardiac involvement was suspected due to (a mixture of) typical clinical symptoms such as frequent palpitations and/or supra- and ventricular premature beats in long-term holter monitoring (n=6), aberrant rest ECG findings including bifascicular blocks (n=5), ventricular tachycardia (n=2), severe or otherwise unexplainable heart failure (n=5) or shortness of breath (n=3). Two patients initially presented with both acute chest pain and elevated cardiac enzymes. Coronary artery disease was excluded in these patients invasively. Two patients had biopsy-proven cardiac involvement and were referred for follow-up examinations.

At the time of imaging, 11/15 patients were treatment-naïve. Patient #5 presented with corticosteroids and azathioprine due to extensive pulmonary involvement, patients #6, #11, and #14 were treated with prednisolone. During follow-up, four patients developed severe ventricular tachycardia that required the implantation of an ICD (n=2) or protection by a life vest (n=2). All other patients had no cardiac events. However, six subjects were started on immunosuppressive therapy (after imaging).

A concise overview of patients′ characteristics can be found in Table 1.

**Table 1:** Patients' characteristics

No.	Age	Sex	Sites ofsarcoidosis	Clinical findings	^68^Ga-DOTATOC	CMR(T2/LGE)	Clinical follow-up
**1**	47	F	LN, lung	Palpitations	negative	negative/negative	unremarkable
**2**	54	F	LN, lung	Bifascicular block, cardiac enzymes, HF, VPB	positive	positive/positive	VT, ICD
**3**	52	M	LN, lung	Cardiac enzymes, chest pain	negative	positive/positive	Initiation of corticosteroids
**4**	41	M	Lung	Palpitations	negative	negative/negative	unremarkable
**5**	52	F	LN, lung	Palpitations, SOB	negative	negative/negative	unremarkable
**6**	51	F	LN, lung, nasal sinus, spleen	Palpitatons, SOB	negative	negative/negative	Initiation of corticosteroids, azathioprine
**7**	75	M	LN, lung	SOB, palpitations, LAFB	positive	positive/positive	Initiation of corticosteroids, azathioprine
**8**	42	F	Bone, LN, liver, lung, spleen	HF, RBBB	positive	positive/positive	VT, cardiogenic shock
**9**	60	F	-	HF, LBBB	negative	negative/negative	unremarkable
**10**	42	M	LN, lung, skin	VT	positive	positive/positive	VT, ICD
**11**	55	F	LN, liver, lung, omentum	HF, LBBB	negative	positive/positive	unremarkable
**12**	65	M	LN, lung, parotidea, uvea	VPB	positive	positive/positive	unremarkable
**13**	52	M	LN, lung	Known CS	positive	positive/positive	unremarkable
**14**	71	F	LN, lung, skin	Known CS	negative	positive/positive	unremarkable
**15**	25	F	LN, lung	VT, HF	positive	positive/positive	VT, lifevest

CS = cardiac sarcoidosis, F = female, HF = heart failure, ICD = implantable cardioverter defibrillator, LAFB = left anterior fascicular block, LN = lymph nodes, M = male, RBBB = right bundle branch block, SOB = shortness of breath, VPB = ventricular premature beats, VT = ventricular tachycardia

### Imaging results

On visual inspection, SSTR-PET/CT returned positive findings in 7/15, CMR in 10/15 patients. Of the 3 CMR^+^/PET^−^ subjects, one patient (patient #3) with minor involvement (<25% of wall thickness in CMR) was missed by PET, most likely due to partial volume effects and the rather modest intensity of the tracer signal. The remaining two CMR^+^/PET^−^ patients (patients #11 and #14) displayed no adverse cardiac events during follow-up, and repeated CMR imaging revealed stable morphologic myocardial changes. In total, both modalities returned concordant results in 12/15 subjects.

**Figure 1:**
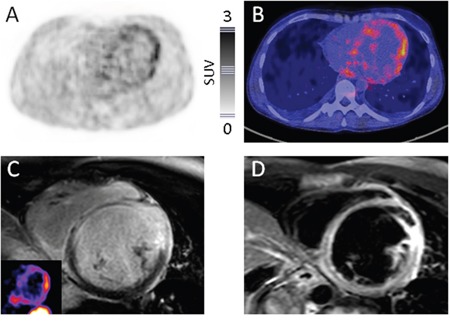
Display of a patient with active myocardial sarcoidosis Consistency of ^68^Ga-DOTATOC-PET and CMR Display of transaxial ^68^Ga-DOTATOC-PET (**A.**, color bar indicating standardized uptake values), fused PET/CT **B.**, short-axis late gadolinium enhanced (LGE) (**C.**, insert: corresponding PET slice), and short-axis T2-weighted CMR **D.** slices in a 42-year-old woman (patient #8) with multi-organ sarcoidosis. PET presents enhanced tracer uptake in large portions of the left ventricle, highly consistent with cardiac involvement of sarcoidosis. CMR yields corresponding results. Of note, the patient experienced sustained ventricular tachyarrhythmia with subsequent cardiogenic shock five days after somatostatin-directed imaging requiring mechanical circulatory support with a percutaneous microaxial blood pump and veno-arterial extracorporeal membrane oxygenation.

**Figure 2:**
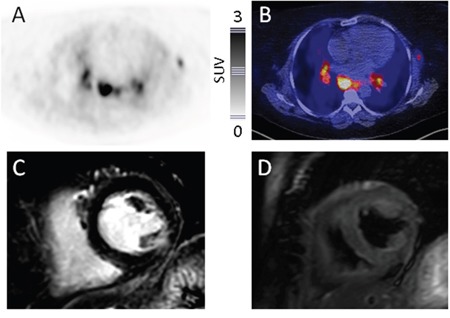
Display of a patient without myocardial sarcoidosis Consistency of ^68^Ga-DOTATOC-PET and CMR Display of transaxial ^68^Ga-DOTATOC-PET (**A.**, color bar indicating standardized uptake values), fused PET/CT **B.**, short-axis late gadolinium enhanced (LGE) **C.**, and short-axis T2-weighted CMR **D.** slices in a 51-year-old woman (patient #6) with multi-organ sarcoidosis. PET presents enhanced tracer uptake in a left axillary and mediastinal lymph nodes, highly consistent with active sarcoidosis. In correspondence to CMR, no signs of myocardial involvement can be recorded. Follow-up is unremarkable regarding cardiac events.

In a total of 255 segments analyzed, there were 27 segments positive in ^68^Ga-DOTA-TOC-PET/CT and 29 segments positive in CMR, respectively. Overall, retention of the radiotracer ^68^Ga-DOTA-TOC was rated mild or moderate and no segment was rated intense (mild retention in 11/27 (41%) segments; moderate retention, 16/27; 59%). Typical PET-findings illustrating enhanced tracer uptake in both a patient with extensive (patient #8) as well as a subject without cardiac involvement (patient #6; positive mediastinal lymph nodes present) are given in Figure 1 and Figure 2, respectively.

On a head-to-head comparison, SSTR-PET and CMR were concordantly positive in 23 segments (23/29 segments positive at CMR; 79.3%). 4 segments were SSTR-PET positive and MRI-negative (1.6%; 4/255), 6 SSTR-PET negative and MRI-positive (2.4%; 6/255). Both modalities returned negative results in 222 segments, thus leading to an overall concordance of 96.1% (Table 2). The individual results of the 17-segment analysis for both sub-groups can be inferred from Supplementary Figure S1.

**Table 2:** Concordance of cardiac MRI and somatostatin-receptor- (SSTR-) based PET/CT in patients with suspected cardiac sarcoidosis

Total collective (n=15)		Cardiac MRI		Σ
		positive	negative	
SSTR-PET	positive	23	4	27
	negative	6	222	228
Σ		29	226	255

### Semi-quantitative analysis

SUV_mean_ and SUV_max_ were significantly higher in the inflamed myocardium as compared to remote myocardium or the left ventricular (LV) cavity. SUV_mean_ in inflamed areas ranged from 1.3-4.5 (mean, 2.0±1.2) and SUV_max_ from 1.4-5.0 (mean, 2.6±1.2), respectively. The SUV_mean_ ratios of lesion-to-remote myocardium were 1.8±0.2 and for lesion-to-LV cavity 1.9±0.2. The corresponding SUV_max_ ratios were 2.0±0.3 and 1.7±0.3, respectively.

## DISCUSSION

To our knowledge, this is the first study to assess the performance of SSTR-directed PET/CT in comparison to CMR in cardiac sarcoidosis. General feasibility of somatostatin receptor targeted imaging of inflammatory cells in sarcoidosis by means of ^111^In-labeled pentetreotide had already been demonstrated in the late 90s [19, 20]. Our group previously reported on the suitability of SSTR-PET/CT in a single patient with septal involvement of sarcoidosis [18]. In this two-center study, we were able to confirm previous results. SSTR-PET/CT identified myocardial uptake consistent with inflammatory activity of sarcoid granulomas in 7/15 patients, thereby demonstrating high concordance with CMR. Distribution of the radiotracer matched with CMR late-gadolinium-enhancement and T2-weighted edema, demonstrating focal accumulation within areas of myocardial inflammation in 23 of 29 involved segments (79%).

On a patient basis, one patient with minor involvement (<25% of wall thickness in CMR) was missed, most likely due to partial volume effects and the rather modest intensity of the tracer signal. The remaining two CMR+/PET- patients displayed no adverse cardiac events during follow-up. Of note, repeated CMR imaging revealed stable morphologic myocardial changes consistent with chronic post-inflammatory processes.

Though tracer uptake in damaged myocardium was almost twice as high as compared to the background of the left ventricular cavity or remote unaffected myocardium, SUV_max_ of sarcoid lesions ranged between 1.4 and 5.0 and were therefore much lower than those reported for ^18^F-FDG [21, 22]. However, since the myocardium does not display any physiologic ^68^Ga-DOTATOC uptake, tracer retention in the heart can be considered specific. Interestingly, in one patient in whom both ^18^F-FDG- and ^68^Ga-DOTATOC-PET were available in addition to CMR (patient #10), ^18^F-FDG failed to detect active inflammation whereas both SSTR-PET and CMR returned findings highly consistent with sarcoidosis. Clinically, the patient developed ventricular tachyarrhythmia the day after CMR.

As another advantage, SSTR-directed imaging obviates the need to adhere to specific patient preparation protocols including prolonged fasting, ingestion of low-carbohydrate, high-fat meals or the injection of heparin prior to radiotracer injection [12, 13, 23].

Up to now, only few studies have addressed the value of SSTR-PET/CT for the imaging of cardiovascular inflammation with most reports focusing on the detection of atherosclerosis [14–16].

In its translation to cardiac sarcoidosis, this approach might allow for direct assessment of disease activity, especially in the course of treatment. While the current gold standard MRI depicts structural changes like cardiac damage and scarring and edema with the highest spatial resolution, ^68^Ga-DOTATOC uptake may directly reflect the underlying immunological cell activity. In future, given the complementary nature of PET and MRI signals, the combination of the two may be the optimal diagnostic approach, preferably by integrated MRI/PET.

Additionally, whereas a recent study comparing ^68^Ga-DOTANOC and ^18^F-FDG PET/CT has demonstrated encouraging diagnostic accuracy for SSTR-directed PET [24], the prognostic value of ^68^Ga-DOTATOC PET/CT, especially in comparison to ^18^F-FDG has to be clarified in future trials. Whereas higher quantitative ^18^F-FDG uptake in CS patients with ventricular tachycardia than in those with atrioventricular block and asymptomatic controls and a higher risk of death or ventricular tachycardia as compared to JMHWG criteria or the ejection fraction have been reported for ^18^F-FDG [9, 25], nothing is known about patient prognostication by SSTR-PET/CT so far. Interestingly, one patient with extensive cardiac involvement (patient #6) experienced sudden malignant ventricular tachycardia with subsequent cardiogenic shock requiring mechanical circulatory support. Although no conclusions can be drawn from a single example in this patient cohort, the extent of SSTR-PET+ myocardial areas might provide the same prognostic information as shown for ^18^F-FDG-PET/CT, given that somatostatin receptor-directed PET directly targets the underlying inflammatory cells.

Our work has several limitations. Most importantly, histopathologic proof of sarcoid granulomas and SSTR expression as the adequate reference test was not available. Cardiac gating for PET imaging was not performed. Early contrast enhancement CMR studies were not included. Data on short-term reproducibility of our findings are missing and should be the subject of subsequent work.

In conclusion, imaging of cardiac sarcoidosis using SSTR-targeted PET/CT is feasible. Our pilot data warrant further analysis in a larger prospective series.

## MATERIALS AND METHODS

### Subjects and study design

A total of 15 patients (6 males and 9 females, median age, 52 years: range, 22-80 years) with histologically proven sarcoidosis (14/15) and the clinical suspicion of cardiac involvement underwent both SSTR-PET/CT as well as CMR on a compassionate use base at the German University Centers of Bonn and Würzburg. No patient had a current or prior history of cardiac disease (e.g. prior infarction, myocarditis or cardiomyopathy). Imaging was performed within a median of 27 days (range, 1-57 days).

German federal laws accept the use of the radiotracer ^68^Ga-DOTA-TOC under conditions of the pharmaceutical law. Written informed consent was obtained prior to the study from all patients.

### Preparation of ^68^Ga-DOTATOC

In Würzburg, ^68^Ga–DOTATOC was prepared using a modification of the method described previously by Breeman et al. [26] using a SCINTOMICS module (Scintomics, Fürstenfeldbruck, Germany). The synthesis was carried out on a computer-assisted synthesis module (Scintomics, Fürstenfeldbruck, Germany). The labeling procedure was optimized concerning amount of peptide, reaction time and reaction temperature. Radiochemical purity was determined by gradient HPLC (Scintomics, Fürstenfeldbruck, Germany).

In Bonn, an improved manual method developed by Eppard and colleagues was used [27].

### PET imaging

PET scans were acquired using an integrated PET/CT scanner (Siemens Biograph 2 [Bonn], Siemens Biograph mCT 64 [Würzburg], Siemens, Knoxville, USA) consisting of a LSO full-ring PET and a 2 or 64-slice spiral CT. 124±31 MBq of ^68^Ga-DOTA-TOC were injected. After a period of 60 min, transmission data were acquired using low-dose CT of the thorax (16 mAs, 130 kV, 512 × 5215 matrix, 5 mm slice thickness, increment of 15 mm/s, rotation time of 0.8 s, and pitch index of 1.2 [Bonn], 80 mAs, 120 kV, 512 × 512 matrix, 5mm slice thickness, increment of 30 mm/s, rotation time of 0.5s, and pitch index of 0.8 [Würzburg]). Consecutively, PET emission data of the heart were acquired in three-dimensional mode over 10 minutes. PET data were reconstructed into a 128 × 128 [Bonn] or 200 × 200 [Würzburg] matrix using the iterative algorithm implemented by the manufacturer including decay and scatter correction based on the acquired CT data.

Images were first inspected visually by two experienced nuclear medicine physicians (CL and AKB; RAB and FCG, respectively). For quantification of increased tracer uptake, a visual score using the terms “mild”, “moderate” and “intense” was used. Areas of increased ^68^Ga-DOTATOC accumulation were documented using the 17-segment AHA heart model [28].

For semi-quantitative analysis, the axial PET image slice with maximum cardiac uptake was selected. A standardized 15mm circular region was placed over the area with the peak activity. This first ROI was used to derive maximum (SUV_max_) and mean standardized uptake values (SUV_mean_). SUV_max_ and SUV_mean_ were also derived in normal reference regions defined by two distinct methods: (1) a second ROI (diameter of 15 mm) in a remote region of the left ventricular wall (not supplied by the culprit vessel) without late-gadolinium-enhancement (LGE) in the corresponding MRI data (if applicable) and (2) another ROI with a diameter of 25 mm in the left ventricular cavity. Signal-to-background ratios were calculated for each method.

### CMR imaging

CMR was performed on a 1.5 T (n=12, Achieva 1.5T, Philips Healthcare, Best, The Netherlands) and 3.0T (n=3, Achieva DS 3.0T, Philips Healthcare, Best, The Netherlands) scanner using dedicated phased array coil for radiofrequency reception. Sequences were gated to the heart cycle via a four lead vector cardiogram. The protocol included a morphologic study based on balanced turbo field echo sequences for documentation of standard cine long and short axis views (FOV 380 mm, flip angle 60°, TE 2.6 – 3.0 ms, TR 130-158 ms). A T2-weighted multi echo gradient echo sequence was used for imaging myocardial edema in both long and short axis (FOV 370 ms, NSA 2, TE 90 ms, TR 2000 -3600 ms, TR (beats) 3). Late enhancement imaging was performed 9 to 12 min after antecubital intravenous administration of 0.15 mmol/kg of a gadolinium based contrast agent (Gadobutrol, Bayer HealthCare, Leverkusen, Germany). An inversion recovery T1 turbo field echo sequence was used, and the inversion time was adjusted to completely null the myocardial signal.

Image analysis was performed using the Extended Workspace software (EWS, Philips Healthcare, Best, The Netherlands). In analogy to PET, all LGE and T2-weighted scans were segmentally analyzed with regards to scar and edema distribution within the myocardium according to the 17-segment model. Concordant signal enhancement in both LGE and T2-weighted sequences was considered as CMR positive for active cardiac involvement. LGE positivity in parallel with T2 negativity was rated as consistent with fibrotic changes.

### Statistical analysis

Most data presented in here are descriptive. Quantitative data are presented as median, range, and mean ± SD.

## SUPPLEMENTARY MATERIALS FIGURE


