# Supplementary material for: Somatostatin receptor based PET/CT in patients with the suspicion of cardiac sarcoidosis: an initial comparison to cardiac MRI
Source: Oncotarget. 2016 Oct 21;7(47):77807–14. doi: 10.18632/oncotarget.12799 (PMC5363622; doi:10.18632/oncotarget.12799)
Supplement: Supplementary file 1 [file oncotarget-07-77807-s001.pdf]

Somatostatin receptor based PET/CT in patients with the suspicion of cardiac sarcoidosis: an initial comparison to cardiac MRI

SUPPLEMENTARY FIGURE

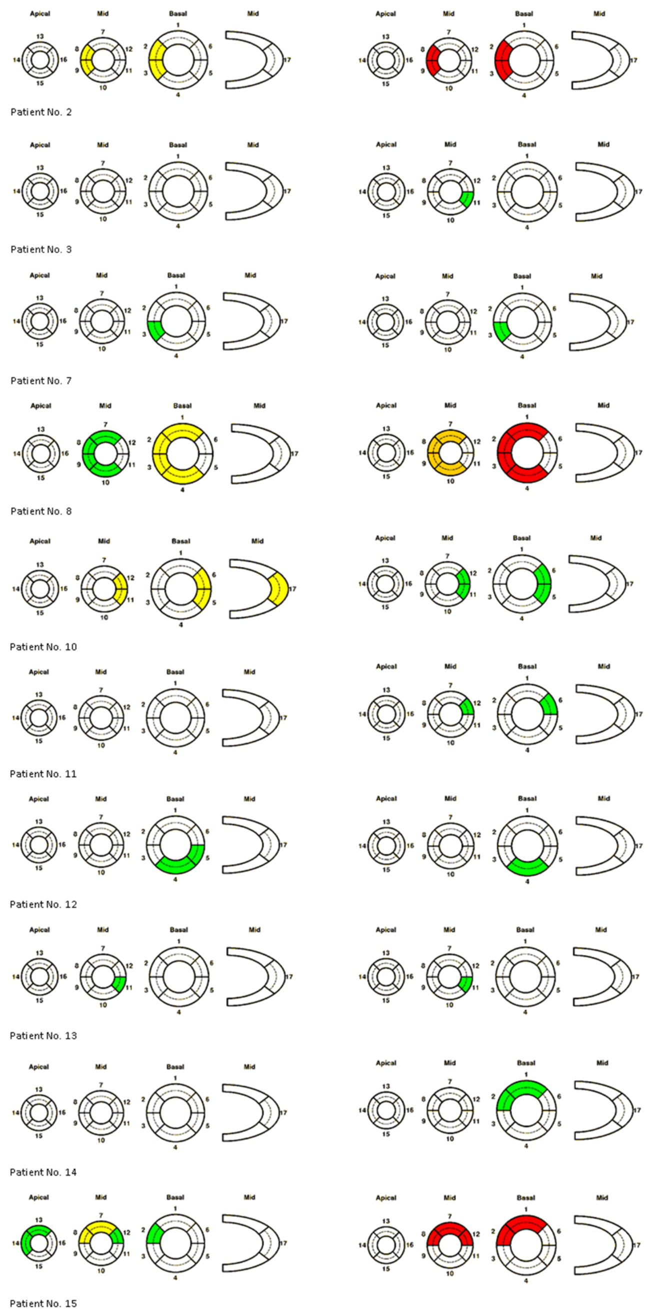

Supplementary Figure S1: Segmental analysis of all patients with positive PET/CMR findings Left side. PET analysis (Color coding for signal intensity: green=mild, yellow =moderate tracer retention; right side: CMR analysis (Color coding for transmurality: white= none, green= <25%, orange = 50 – 75%, red = 75- 80%).
